# A comparison of elasticities of viral levels to specific immune response mechanisms in human immunodeficiency virus infection

**DOI:** 10.1186/1756-0500-7-737

**Published:** 2014-10-20

**Authors:** Sarudzai P Showa, Farai Nyabadza, Senelani D Hove-Musekwa, Gesham Magombedze

**Affiliations:** Department of Applied Mathematics, National University of Science and Technology, P.O. Box AC 939 Ascot, Bulawayo, Zimbabwe; Department of Mathematical Sciences, University of Stellenbosch, P. Bag XI, Matieland, 7602 South Africa; National Institute for Mathematical and Biological Synthesis, University of Tennessee, 1122 Volunteer Blvd, Knoxville, TN USA

**Keywords:** HIV immune responses, Elasticity analysis, Discrete time models

## Abstract

**Background:**

The presence of an asymptomatic phase in an HIV infection indicates that the immune system can partially control the infection. Determining the immune mechanisms that contribute significantly to the partial control of the infection enhance the HIV infection intervention strategies and is important in vaccine development. Towards this goal, a discrete time HIV model, which incorporates the life cycle aspects of the virus, the antibody (humoral) response and the cell-mediated immune response is formulated to determine immune system components that are most efficient in controlling viral levels. Ecological relationships are used to model the interplay between the immune system components and the HIV pathogen. Model simulations and transient elasticity analysis of the viral levels to immune response parameters are used to compare the different immune mechanisms.

**Results:**

It is shown that cell-mediated immune response is more effective in controlling the viral levels than the antibody response. Killing of infected cells is shown to be crucial in controlling the viral levels. Our results show a negative correlation between the antibody response and the viral levels in the early stages of the infection, but we predicted this immune mechanism to be positively correlated with the viral levels in the late stage of the infection. A result that suggests lack of relevance of antibody response with infection progression. On the contrary, we predicted the cell-mediated immune response to be always negatively correlated with viral levels.

**Conclusion:**

Neutralizing antibodies can only control the viral levels in the early days of the HIV infection whereas cell-mediated immune response is beneficial during all the stages of the infection. This study predicts that vaccine design efforts should also focus on stimulating killer T cells that target infected cells.

**Electronic supplementary material:**

The online version of this article (doi:10.1186/1756-0500-7-737) contains supplementary material, which is available to authorized users.

## Background

The human immune system is a complex network of cells, chemicals and organs that keeps an individual healthy. There are three lines of defense that make up the human immune system. The three types of immunity are, passive immunity, innate/non-specific immunity and adaptive/specific immunity. Passive immunity is borrowed from an external source and lasts for a short time. Innate immunity comprises of barriers such as the skin and the mucous membranes. There are two major branches of the specific immune responses which are the humoral immune response and the cell-mediated response. Humoral immunity is mediated by B cells and cell-mediated immunity involves the production of cytotoxic T-lymphocytes (CTLs), activated macrophages, activated natural killer (NK) cells and cytokines in response to an antigen and is mediated by T-lymphocytes.

Understanding the interactions of these immune system mechanisms during an HIV infection is of great importance in HIV treatment and vaccine development. However, there are no good animal models for this infection and mathematical models have been the basic tool used to understand these interactions [1–4]. All the models developed and the related HIV research pointed to the same conclusion that, although the immune response poses a tough challenge to HIV infection, the virus adopts several immune system escape mechanisms. The virus can escape the immune system through mutations [5–7], the formation of the viral latent reservoir [8, 9] and through the use of its proteins such as the *Vif*, *Vpu*, *Tat* [10] and *Nef* [11]. Treatment regimes were developed to augment the human immune system in the fight against HIV infection. However, these treatment regimes are not easily accessible in developing countries and hence the need to find a cure or vaccine for the infection.

In this study, we model the within host dynamics of the HIV infection using discrete time models because of their relative simplicity in computing transient elasticities of viral levels to immune system parameters compared to ordinary and partial differential equations (ODEs and PDEs). A discrete time model also allows the incorporation of all the life cycle aspects of the virus and yet remain relatively simple to analyse compared to ODEs and PDEs. The main advantage of transient analysis over asymptotic analysis is that it focuses on perturbations to the population structure rather than perturbation analysis on demographic rates only [12]. Transient elasticity analysis is also effective in analyzing the effects of changes of parameters and initial conditions on population levels in the short term.

Ecological modelling tools are employed to model the interplay between the immune system and the HIV pathogen. The human immune system is treated as an ecosystem and the components of the immune system are treated as species in that ecosystem. Pathogens, in this case, HIV, are then defined as exotic species, which can either invade the ecosystem or be driven to extinction. In an ecosystem, all organisms are connected by ecological relationships namely predation, competition, mutualism, commensalism, amensalism and parasitism. Such relationships are comparable to the human immune system. In the human immune system, examples of such relationships include CTLs hunting and killing infected cells, antibodies neutralizing viruses, one strain of HIV competing for CD4 ^+^ T cells, two or more HIV strains competing for CD4 ^+^ T cells, CD4 ^+^ T cells offering a catalytic effect to both the B cells and the CD8 ^+^ T cells and immune surveillance, where different components of the immune system interact to maintain homeostasis. Transient elasticity analysis is then used to compare the two arms of the specific immune response and hence inform on HIV vaccine or treatment development. Elasticity analysis can be defined as a method of evaluating how proportional changes in model parameters affect the population growth. Parameters with high absolute values of elasticities are predicted to give greatest changes in population levels when altered by a fixed quantity, thereby predicting good targets for the control. In this study, the immune response in which these parameters are found, is the immune response that is predicted to be most effective in controlling the viral levels.

## Results

### Model simulations

Numerical simulations are performed to show the dynamics of HIV infection under different immune response mechanisms. These simulations are performed in order to identify the specific immune system component that is most effective in controlling the HIV infection. Each immune response mechanism is considered separately by setting all the other immune response mechanisms to zero, except the ones under consideration. For instance, to consider the chemokine antiviral response which work by reducing the infectivity of the virus, the expressions labeled antibody, lytic and cytokine are set to 1 in the model which include specific immune responses (system of equations (7)-(14)). This model (with chemokine response only) is called the chemokine response model in this study. The model with non zero immune system parameters is referred to as the combined model. The basic model is the resulting model after all the immune system parameters are set to zero (the system of equations (1)-(6)). The function of the basic model is to act as a control, in the sense that it gives the viral, infected and uninfected cell levels, before the intervention of the immune system.

We carried out literature search to obtain parameters we used to carry out simulations. Where we could not find parameters values, we used values that could generate acceptable HIV dynamics. The parameter value for *β*_1_ was obtained from [13] and a description of how this parameter value was computed is also given [13]. It was found by a study in [14] that the infectivity of the cell associated virus is 10^2^ to 10^3^ times greater than the infectivity of free virus stocks so we multiplied the infectivity of the free virus by values in the ranges 10^2^ to 10^3^ to get the infectivity of the cell-associated virus *β*_2_. However, this range is from an in vitro model. In order to find the form of transmission that is more efficient in the blood and support the use of the values obtained from an in vitro model, we conducted a study using our mathematical models to find the form of transmission that is more efficient in vivo. Results showed that cell-to-cell transmission was efficient in transmitting the infection than cell-free transmission, a result consistent with in vitro models [15–17]. Part of the results of this study are given in Additional file 1. A comprehensive sensitivity analysis of immune response parameters is performed in the elasticity analysis section. Sensitivity analysis of the viral levels to the viral life cycle parameters is added in Additional file 1. The parameter values used for simulations are given in Table 1.

**Figure 1:**
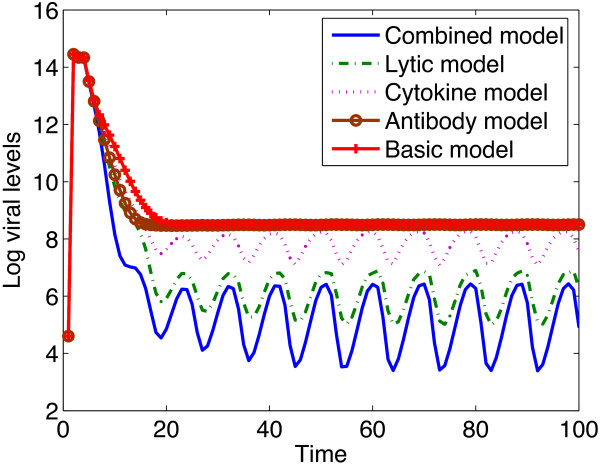
**Plots of viral levels against time under different immune response models.** Neutralizing antibodies are not very efficient in controlling viral levels. The antibody response model yielded levels that settled to levels comparable to those of the basic model (model without immune system intervention). Lowest viral levels are obtained when all the specific immune system components work simultaneously as is shown by the graph of the combined model. Killing of infected cells also play a very crucial role in reducing the viral levels as can be seen from the graph of the lytic model. The parameter values used are given in Table 1 and the initial conditions were .

Numerical simulations of the viral levels against time under the different immune response models are shown in Figure 1.

Simulations of models with immune responses resulted in reduced viral levels, though the reduction was high when all the immune system’s antiviral responses were combined. The chemokine immune response model predicted viral levels that are comparable to those of the basic model. The graph of the chemokine immune response model was so close to that of the basic model so that they appeared as overlapping graphs and was therefore omitted. We can therefore predict that chemokines are not efficient in controlling viral levels. During the early days of the infection, the reduction in viral levels due to the neutralizing antibody response model was comparable to that of the combined response model, however as the infection progresses, it became comparable to the basic model. This result suggests that neutralizing antibodies reduce the viral levels significantly in the acute stage of this infection and that their role diminishes with infection progression. It can also be seen from Figure 1 that lysing of infected cells is critical in controlling the viral levels.

**Table 1:** **Parameter values**

Parameter	Description	Value	Source
*β*_1_	Virus infectivity	0.000024 *m* *l* ^−1^ *d* ^−1^	[13]
*β*_2_	Cell-associated	100−1000×	[14, 15, 17]
	Virus infectivity	0.000012 *m* *l* ^−1^ *d* ^−1^	
*h*_1_	Neutralizing efficiency	0.001 *m* *l* ^−1^ *d* ^−1^	Est.
*h*_2_	CTLs killing efficiency	0.01 *m* *l* ^−1^ *d* ^−1^	Est.
h	Cytokine killing efficiency	0.0011 *m* *l* ^−1^ *d* ^−1^	Est.
*μ*_*T*_	Death rate of CD4 ^+^ T cells	0.02 *d* ^−1^	[18]
*ω*	Saturation constant	0.01 *m* *l* ^−1^	Est.
*S*_*T*_	Source term for CD 4^+^ T cells	10 cells *d* ^−1^	Est.
*μ*_*C*_	CTL death rate	0.5 *d* ^−1^	Est.
	Infected cell death rate	0.5 to 1 *d* ^−1^	[18]
*μ*_*B*_	B cell death rate	0.5 *d* ^−1^	Est.
*θ*_1_	Transition probability	1/3	[19]
*θ*_2_	Transition Probability	0.06315	Computed
*θ*_3_	Transition Probability	0.43685	Computed
*ϕ*	Viral production/cycle/provirus	1000	Est.
*ψ*	Probability of proliferation of CTLs	0.01	Est.
*χ*	Probability of proliferation of B cells	0.01	Est.
*a*_1_	Saturating constant	0.002 *m* *l* ^−1^	Est.
*a*_2_	Saturating constant	0.002 *m* *l* ^−1^	Est.

Parameters values that were not obtained from literature were chosen to be in the ranges 0 to 1. Est. means parameters were estimated/derived to simulate acceptable HIV dynamics. The infectivity of the cell associated virus is 10^2^ to 10^3^ times greater than the infectivity of free virus stocks [14], so we multiplied the infectivity of the free virus by values in the ranges 10^2^ to 10^3^ to get the infectivity of the cell associated virus.

**Figure 2:**
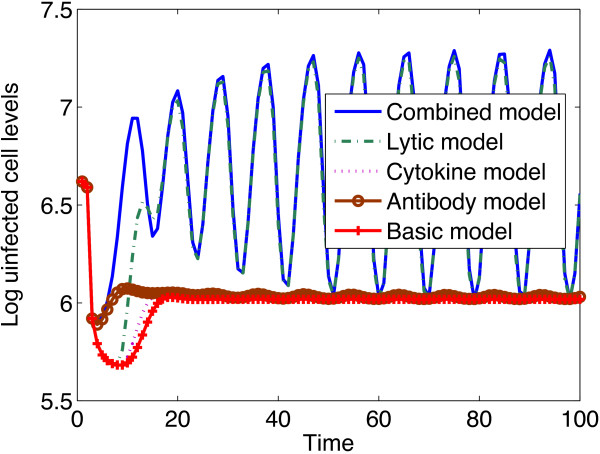
**Plots of uninfected CD4** ^**+**^ **T cell levels against time under different immune response models.** All immune response models predict increased uninfected CD4 ^+^ T cell levels, with the highest increase predicted from the combined model. Cytokines and chemokines are inefficient in maintaining high uninfected cell levels. The graphs of the cytokine and chemokine models are shown to settle to values close to those of the basic model. The parameter values used are given in Table 1 and the initial conditions were .

Simulations of uninfected CD4 ^+^ T cell levels under the different immune response models are shown in Figure 2, in which uninfected CD4 ^+^ T cells are demonstrated to increase, with the highest increase achieved with the combined immune response model.

From Figure 2, it can be observed that the role of the antibody response in maintaining high uninfected cell levels is more defined during the early days of the infection than during the later days of the infection. The cytokine and the chemokine responses are not effective in maintaining high uninfected cell levels. The graph of the chemokine response model coincided with the graph of the basic model. It can also be seen that the lytic antiviral response play a significant role in maintaining high uninfected cell levels.

**Figure 3:**
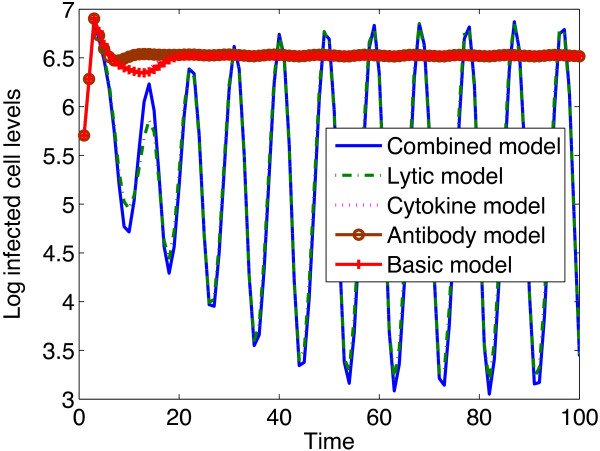
**An illustration of simulated infected CD4** ^**+**^ **T cell levels with different immune response models.** Infected CD4 ^+^ T cell levels from the cytokine and neutralizing antibody models settle to levels that are comparable to those of the basic model (a model without immune system intervention). The parameter values used are given in Table 1 and the initial conditions were .

Figure 3 shows infected cell levels obtained from different immune response models. The cytokine and antibody models settled to levels comparable to those of the basic model, though the levels are slightly higher than those from the basic model. The lytic antiviral response model resulted in the lowest infected cell levels. The combined models predicts infected cell levels that are comparable to those of the lytic response.

The viral, infected and uninfected cell levels show an oscillatory behavior even if the time (*t*) is increased to 200. This is a common feature of the predator-prey models that we have used.

### Elasticity analysis

**Figure 4:**
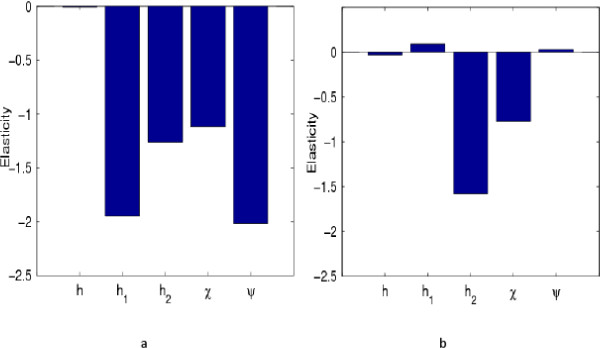
**Elasticities of the viral levels to immune response parameters.** **a**, gives the elasticities plot at time 10. Increases in all parameter values result in reduced viral levels. However the chemokine antiviral response which is represented by the parameter *h*, does not significantly contribute to the control of viral levels. Parameters from the antibody response (*h*_1_,*ψ*), are predicted to be the best targets for the viral level control. **b**, gives the elasticities plot at time 30. Parameters from the antibody response have positive elasticity values and thus their increases result in increased viral levels. The lytic antiviral response is predicted to be the best target for the control of viral levels. The parameter values used are given in Table 1 and the initial conditions were .

Transient elasticity analysis of the viral level to immune system response parameters which is another way of performing sensitivity analysis was done using the methods suggested in [20], to substantiate and clearly demonstrate the influence of immune response parameters on viral levels. The sensitivity of the viral level to immune response parameters is given by  and the elasticity of the viral level to immune response parameters is given by  where *θ* is a vector containing the immune response parameters. The derivation of this formula is given in Additional file 2. A negative elasticity value means that there is an inverse relationship and a positive elasticity means that there is positive correlation. Elasticity values at time point 10 and time point 30 are given in Figure 4. The graphs show that the elasticities of the viral levels to immune response parameters are time dependent.

**Figure 5:**
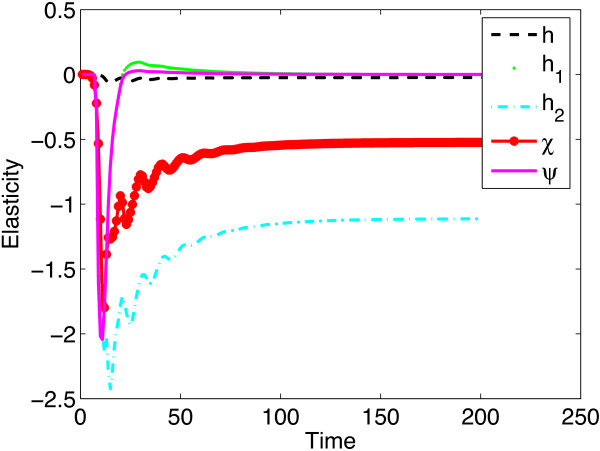
**Transient elasticities of the virus population with respect to immune system parameters.** During the early days of the infection the viral level is more elastic to the neutralizing antibody response parameters but as the infection progresses the viral levels are more elastic to the cell-mediated response parameters. The elasticities of the viral level to the antibody response parameters changed from negative to positive as time progressed. Thus we can conclude that neutralizing antibodies are only efficient in controlling the viral levels in the early days of the infection. The parameter values used are given in Table 1 and the initial conditions were .

In Figure 5, we give the transient elasticity values over 200 time points.

It was observed that during the early days of the infection the viral level was most elastic to the probability of proliferation of B cells *ψ*, followed by *h*_1_, the neutralizing efficiency of antibodies. Since these elasticity values are negative, it means that if the probability of proliferation of B cells and the neutralizing efficiency of antibodies are increased as soon as the virus is introduced, lower viral levels will be obtained. Since these two parameters are from the neutralizing antibody response, it means that the neutralizing antibody response is more effective in controlling the viral levels than any other specific immune component during the early days of the infection. However, as time progressed, the viral level was most elastic to the lytic antiviral response parameter *h*_2_, followed by the probability of proliferation of CTLs, *χ* and was least elastic to *h*, the cytokine antiviral response parameter. This means that killing of infected cells is most effective in controlling the viral levels as soon as the infection become established, followed by the probability of proliferation of these CTLs. The elasticities of the viral level to the antibody response parameters changed from negative to positive as time progressed and these positive values are very close to zero. Thus we can predict that neutralizing antibodies are only efficient in reducing the viral levels in the early days of the infection and that increasing antibody response parameters once the infection become established will result in elevated viral levels. Elasticity analysis of the viral levels to immune response parameters predicted results that were also obtained by model simulations (see Figure 1). Elasticity analysis of the viral levels to other model parameters is given in Additional file 1.

### Correlations of the viral levels to the immune system components predicted to be efficient in controlling the viral levels

**Figure 6:**
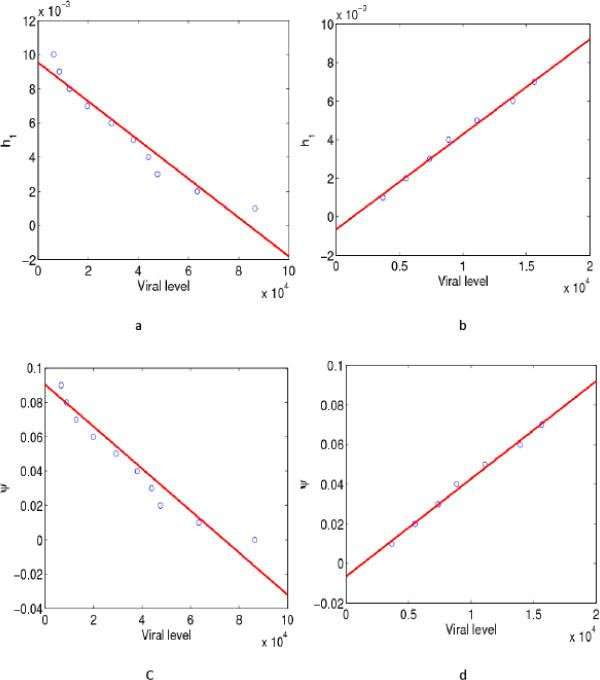
**Scatter plots with model fits between the viral levels and immune response parameters.** **a** and **b** give the scatter plots with linear fits between the viral levels and the neutralizing efficiency *h*_1_ of antibodies at time *t*=10 and *t*=100, respectively. The circles are the data points and the line is the linear fit. In Figure 6 **a**, a negative relationship between the viral levels and the antibody neutralizing efficiency is observed. Figure 6 **b** shows a positive relationship between the viral levels and the neutralizing efficiency. **c-d** give scatter plots with linear fits (correlations) between the viral levels and the probability of proliferation of B cells *ψ*, at time *t*=10 and *t*=100, respectively. Figure 6 **c** shows a negative relationship between the viral levels and the probability of proliferation of B cells. In Figure 6 **d**, a positive relationship between the viral levels and the probability of proliferation of B cells is observed. The parameter values used are given in Table 1 and the initial conditions were .

Results from previous sections show that neutralizing antibodies are only efficient in reducing the viral levels during the early days of the infection and that as time progresses their role changes from decreasing the viral levels to be positively correlated with the viral levels. To visualize these results, we fixed a time point in the early days of the infection and a time point in the late days of the infection then vary the parameters *ψ* and *h*_1_ and plot the viral levels against these parameter ranges in Figure 6.

At time 10, we observed a negative correlation between *h*_1_, the neutralizing efficiency of antibodies and viral levels, and between the probability of proliferation of B cells and viral levels. At time point 100, it can be seen that as the values of *h*_1_ and *ψ* increase, the viral levels also increase. The scatter plots clearly show that the role of antibody (humoral) response depends on the stage of the infection.

**Figure 7:**
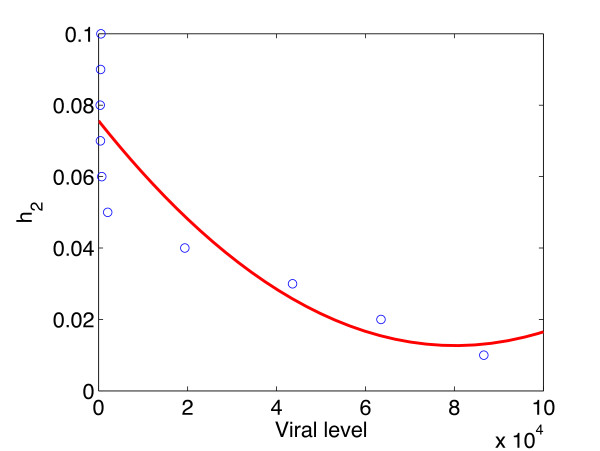
**Scatter plots with a quadratic fit between the viral levels and the infected cell killing efficiency of CTLs.** The red line represent the quadratic fit and the small circles represent the data points (model outcomes). There is a negative relationship which can be best be explained by a quadratic function between the viral levels and the killing efficiency of infected cells by CTLs. The parameter values used are given in Table 1 and the initial conditions were .

We also observed that the viral levels were most elastic to *h*_2_, the killing efficiency of CTLs during the late days of the infection and that the effect of this parameter was not dependent on the stage of the infection (all the elasticity values were negative in Figure 5). This means that an inverse relationship will be observed at all the stages of the infection. We randomly picked time point 100, to generate the scatter plot in Figure 7. The scatter plot in Figure 7 shows that there is a negative correlation between the killing efficiency *h*_2_ and the viral levels. We expect the same relationship at all time points.

### Effects of transmission parameters on viral levels

**Figure 8:**
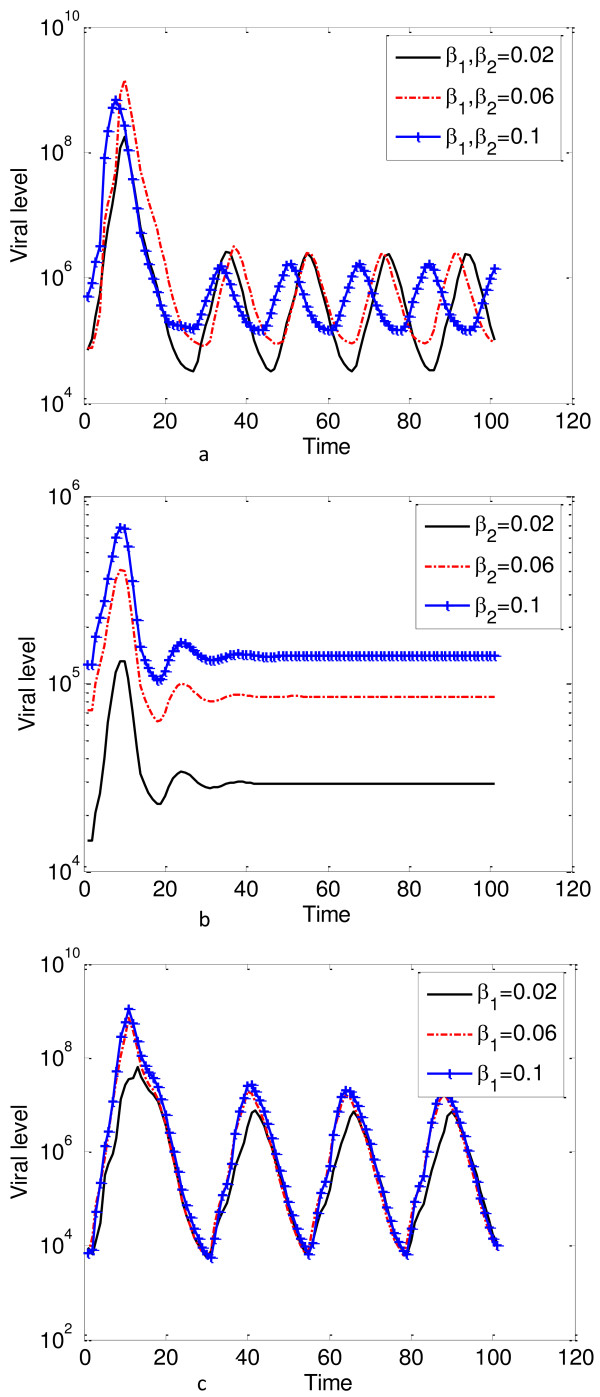
**Effects of the transmission parameters on viral levels.** The plots gives natural log of the viral levels against time. **a** gives the plots for viral levels when *β*_1_ and *β*_2_ are varied simultaneously, **b** gives the plots for viral levels when *β*_2_ is varied and **c** gives the plots for viral levels when *β*_1_ is varied. The viral level increases with increases in *β*_1_ and *β*_2_, however, increasing *β*_2_ had a greater impact on viral levels when compared to increases *β*_1_. The viral levels are more sensitive to *β*_2_ than *β*_1_.

We varied the parameters *β*_1_ and *β*_2_, to see their effects on viral levels and the results are shown in Figure 8.

We observed that the viral levels increase with increases in *β*_1_ and *β*_2_. However, increasing *β*_2_ had a greater impact on viral levels when compared to increases in *β*_1_, and thus we can conclude that the viral levels are more sensitive to *β*_2_ than *β*_1_. When these two parameters were increased simultaneously, after an initial increase, the viral levels will start to decrease with increases in these two parameters. Theoretically, these results imply that the transmission parameters are bifurcation parameters. Increases above certain thresh hold values will impact negatively on viral levels.

## Discussion

Discrete time HIV models that incorporate the life cycle aspects of the virus, the antibody (humoral) response and the T-cell response were formulated to determine immune system components that are most efficient in controlling viral levels. The interplay between the immune system components and the virus at the different stages of its life cycle was modelled using ecological relationships. The flexibility of the developed models allowed an in-depth examination of the interactions between the specific immune system’s antiviral response and the HIV pathogen.

Neutralizing antibodies were predicted to be effective in controlling viral levels in the early days of the infection, a result observed in several experimental studies [21–25]. However, neutralizing antibodies were not able to control the viral levels once the infection become established as was observed in the studies [26, 27]. The reason for this could be the fact that antibodies (HIV predators) neutralize free virus (prey) thereby reducing the parasitoid (HIV) pressure on the host (CD4 ^+^ T cells). This will allow the host population to grow. Reduced levels of free virus means less stimuli and hence reduced levels of B cells. The virus population will then start to grow (due to increased levels of CD4 ^+^ T cells) with two advantages; first, more CD4 ^+^ T cells (host) and second, less B cells (predator). Increases in viral levels are at the expense of CD4 ^+^ T cell levels. When the B cells start to increase due to increased viral levels (prey), they do so with a disadvantage of low CD4 ^+^ T cell levels and hence their proliferation is impaired. It is also important to note that neutralizing antibodies target the early stage of the HIV life cycle, a stage at which viral levels were shown to be least sensitive to [28–30]. Currently there is no information besides the high viral mutation rates, that explain why neutralizing antibodies fail to control the infection. This result provide this understanding and prediction that could be critical in the knowledge required for the designing of effective antibody based vaccines.

The chemokine response model predicted viral levels which settled to the same levels as those of the basic model (a model without immune system intervention that was used as a control). The reason for this could be that, chemokines target the early stages of the HIV life cycle and hence are not effective in controlling viral levels. The lytic antiviral response was predicted to be most effective in controlling the viral levels amongst the three cell-mediated immune responses considered in this study.

A model which combines the neutralizing antibody and cell-mediated immune responses on HIV infection dynamics was also considered. This model demonstrates increased levels of uninfected CD4 ^+^ T cells and reduced viral and infected cell levels. This may have come as a result of the fact that the presence of neutralizing antibodies increases the CD4 ^+^ T cell levels and thereby improving the CTL antiviral response.

Transient elasticity analysis of the viral level to the immune system’s antiviral response parameters was performed. It was observed that during the early days of the infection the viral level was more elastic to neutralizing antibody response parameters than the cell-mediated response parameters, thus increasing the neutralizing antibody response parameters at the sites of HIV entry may circumvent the infection better than the T cell response parameters. This result is logical given the fact that T cell based immune response is elicited by infected cells thus they will have to work when the infection become established. However, it should be noted that although the neutralizing antibody effect resulted in reduced viral levels in the early days of the acute phase, this was at the expense of increased infected cell levels. The elasticities of the viral levels to neutralizing antibody response parameters changed from negative to positive after a few time points. Thus their effect as the disease progresses will change from decreasing the viral levels to increasing the viral levels. From this study we can predict that cell-mediated immune response cannot protect against infectious challenge but can control an established infection whereas the neutralizing antibody response can protect against challenge but can not control established infections.

The elasticities of the viral levels to the cell-mediated immune response parameters are negative for all time points. This result agrees with experimental results that CTLs are effective at all stages of the infection from the acute phase [31–34] to the late days of the infection [35, 36]. We therefore predict that cell-mediated immune response is more effective in controlling the viral levels than the neutralizing antibody response. This result is consistent with experimental results [37–40]. Amongst the four specific immune response components considered (neutralizing antibody, CTLs’ chemokines, cytokine and lytic responses), we found that the CTLs’ lytic antiviral response was most effective in reducing the viral levels. However, from the model structure, it seems that this conclusion is mainly because it is assumed that neutralizing antibodies and chemokine responses do not prevent cell-to-cell spread of HIV at all. Nevertheless, if we were to assume that neutralizing antibodies and chemokines inhibit cell-to-cell transmission thereby reducing the transmission parameter *β*_2_, we will still find that these responses’ effect on viral levels will still be lower than that from the lytic response as these immune responses will be targeting the early stages of the viral life cycle, stages in which it was shown by several studies that the viral levels are least sensitive to [41–44].

Sensitivity analysis of the viral levels to other model parameters was done and details are in Additional file 1. It was shown that the viral levels are most elastic to *ϕ* followed by *θ*_2_ and least elastic to *β*_1_. The implication for this result is that the viral levels are most sensitive to viral production per cell per unit time and least sensitive to the rate at which cells are infected by cell free virus.

Although our model provide predictions that can be used to inform on HIV vaccine and treatment development, it did not include all the specific immune responses such as the antibody-dependent-cellular-cytotoxicity (ADCC) and antibody-depended-cell-mediated-virus-inhibition (ADCVI). Including these antibody effector mechanisms may bring more insights on the role of antibodies in an HIV infection. Moreover, most of HIV replication occurs in the lymph nodes and the lymphatic tissues and not in the blood. In this study replication in the blood was considered, with the hope that if replication in the blood is reduced then the livelihoods of infected persons will improve just as is the case of HIV treatment. Antiretroviral therapy can reduce the HIV viral levels to undetectable levels in the peripheral blood but its effect on the lymphoid tissue is very limited as predicted by failure by most drugs to penetrate the lymphoid tissue and stop the ongoing replication in these tissues [45]. However antiretroviral therapy improve the livelihoods of infected persons despite this loop hole.

HIV-infected cells continue to make viruses in lymphoid tissues even if an individual is under treatment and having undetectable viral levels in the blood [45]. If HIV replication in these tissues can be stopped then HIV can be cured [46]. Modelling HIV replication in the lymphoid tissue will help identify an immune response that is most efficient in controlling replication in this site where the bulk of HIV replication and pathogenesis occurs and this finding may have many potential implications for the future of HIV therapy and for attempts to try and cure HIV infection but is however impeded by lack of data in this compartment. In humans, blood is commonly monitored to measure disease progression and assess immune status and hence infection parameters in blood are readily available than in tissues.

## Conclusion

The CTLs’ lytic antiviral response was predicted to be the most effective in reducing the viral levels during all stages of the infection. It was also observed that the killing of infected cells plays a very significant role in increasing the uninfected cell levels and decreasing the infected cell levels. We therefore predict that concentrating on antibody-dependent-cell-mediating cytotoxicity may be more beneficial in the control of the HIV infection than focussing on neutralizing antibodies only. Another striking result that was observed was that, the effect of neutralizing antibodies on viral levels is depended on the stage of the infection.

## Methods

In this section we develop our models: (i) the basic model considers the interaction between the virus and CD 4^+^ T cells (ii) the specific immune response model is an extension of the basic model that includes the specific immune responses. The models represent cell interaction in the blood compartment where perfect mixing of cells and the virus is assumed. Age structure of infected cells maybe added to the model to get a system of integrodifference equations in the case where age is a continuous variable or a discrete multi-state model in the event where age is discrete. Segregating infected cells by age of infection may bring useful insights but at the expense of a complex model which might be difficult to analyze and may hence fail to meet the objectives of this study. We therefore ignored the age structure of infected cells and assume that infected cells are indistinguishable from each other.

### The basic model

The stages in the HIV replication cycle are; receptor binding, cell entry, uncoating, reverse transcription of viral RNA into DNA, nuclear entry, integration of the viral DNA into the host DNA and transcription and translation of viral RNA, assembly of virus progeny particles and budding. Receptor binding, cell entry, uncoating and reverse transcription of viral RNA into DNA are combined into a single stage, HIV-DNA stage, (*D*). Nuclear entry and integration of the viral DNA into the host DNA are combined into the provirus stage, (*P*). Transcription, translation of viral RNAs and assembly of virus progeny particles are combined to yield the virus progeny. Here, it is assumed that all the virus progeny will mature and thus mature virus production per infected cell per replication cycle is equated to the progeny virion production per replication cycle. This type of stage classification is motivated by the following facts:

- ● Treatment of HIV/AIDS available targets the virus at these stages,
- ● The immune system also targets the virus at these same stages [47–49].
- ● The transition probabilities (proportions that move from one stage to the next stage) at these stages are well defined [50–53].

The duration of the DNA stage is 0.5 days [54] and the provirus stage takes variable time mainly because of the complex nature of the mechanisms involved in the processes of transcription and translation, and the highly probabilistic nature of these processes. We approximate the provirus stage duration by a Negative Binomial Distribution, as illustrated by [55], the details are given in Additional file 2. This resulted in two pseudo-provirus stages with transition probability *θ*_2_=0.5 and the probability that the provirus will survive and remain in the same pseudo provirus stage *θ*_3_=0.43685. We let *D*_*t*_ be the population (level) of viral DNAs, *P*_*t*_ be the provirus population in the pseudo provirus stage 1, *Q*_*t*_ be the provirus population in the pseudo provirus stage 2, *V*_*t*_ be the virus population (viral level), *T*_*t*_ be the CD4 ^+^ T cell population (level) and  be the infected cell population (level). HIV is transmitted through cell free diffusion and cell-to-cell transfer [56, 57], the later being a significantly more efficient mode of transmission [14, 15, 58]. The relationships between a healthy CD4 ^+^ T cell and the virus and between an infected cell and a healthy CD4 ^+^ T cell are modelled using host-parasitoid interactions (Poisson probability distribution) with a slight modification that in host-parasitoid models, there is an assumption that once the host is parasitized (CD4 ^+^ T cell is infected), it is functionally dead until the parasitoid (virus) ‘offspring’ emerge from it. In an HIV infection, there is a time delay before death actually occurs. This results in a mixed population of infected (unparasitised) and uninfected cells (parasitised). The average number of virus attached to a CD 4^+^ T cell is given by . It is assumed that all the virus that manage to attach to the cell will undergo the process of fusion and transcription so that  represents the amount of viral DNA that enter the cell per time step. It has also been observed that almost all HIV linear unintegrated DNA are rapidly transported to the nucleus where they are either processed into two types of cycles or integrated [50]. Only those that are integrated are involved in the HIV replication cycle [59]. Those that circularize can be available at the next time step, but are assumed to be no longer participating in the replication cycle and are therefore are left out in the model. Thus if D does not integrate and become P at time *t*, it is assumed that it will not be available at time *t*+1.The time step for the model is 0.5 days, time spend in the DNA stage. The stage structured model for HIV, without the intervention of immune components, takes the form
123456

This model is referred to as the basic model in the rest of the manuscript. Equation (2) represents the amount of provirus in the first pseudo provirus stage. The proportion of the DNA that survive and grow from the D stage to the P stage is given by *θ*_1_ and the proportion of the provirus that survive and remain in the P stage is given by *θ*_3_. Equation (3) represents the second pseudo proviral stage. The proportion of the provirus that survive and grow from the P to the V stage is given by *θ*_2_, and *θ*_3_ is the proportion of the provirus that survive and remain in the *Q* stage. Equation (4) is the equation of the mature virus population, *ϕ* represents the number of virus particles produced per provirus per replication cycle. Viral production depends on the density of the infected CD4 ^+^ T cells. The parameter *θ*_2_ represents the transition probability from the provirus stage to the virus stage. The same parameters *θ*_2_ and *θ*_3_ were used in equations (2)-(4) because the pseudo stages are assumed to be identical, so the transition and survival probabilities in these stages are the same. We have assumed that the viral level (density) at time *t*+1, does not depend on the viral level at time *t*, because plasma virus have a mean life span of 0.3 days [60] and the time step for the model is 0.5 days, meaning that no plasma virus is able to survive to the next time step.

**Figure 9:**
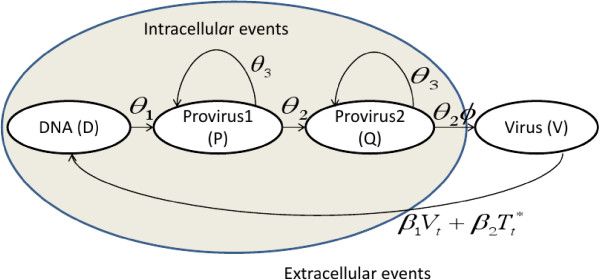
**The life cycle graph for HIV showing the intracellular and the extracellular stages of the virus life cycle.** The provirus stage P, has been broken down into two pseudo stages, *P* and *Q*. The provirus stage duration distribution is approximated by a Negative Binomial Distribution.

Equations (1)-(3) give the intracellular equations of the virus life cycle. The quantities give the levels per cell and to get the total quantities per ml of blood, we multiply the intracellular levels by the number of infected cells per ml, . Equation (4) gives the extracellular equation of the viral life cycle. The expression *θ*_2_*ϕQ*_*t*_ gives the viral production per cell so that  gives the levels per ml of blood. The life cycle graph of the virus showing the interaction of the intracellular and extracellular events is given in Figure 9.

Death of infected cells is modelled through the parameter . Through this parameter, the intracellular steps associated with the dying cells are flashed out of the system when death of infected cells occurs. For this reason we keep track of the remaining infected cells by the term , which is then considered in the intracellular compartment. If one is to kill all infected cells, at time t, there will be zero populations at the intracellular stages at time *t* since the intracellular levels will be multiplied by zero to get the levels per ml.

Equation (5) models the number of CD4 ^+^ T cells at time *t*+1, *S*_*T*_ is the constant supply from the thymus which is assumed to occur at the beginning of the time step and *ν*=1−*μ*_*T*_, where *μ*_*T*_ is the proportion of uninfected cells that die per time step. Death is assumed to occur at the end of the time step so that 1−*μ*_*T*_, gives the proportion of uninfected cells that survive per time step. Uninfected cells must survive infection by infected cells and the virus for them to remain healthy. We have assumed that a CD4 ^+^ T cell can be infected by free virus particles or by cell associated virus through contacts of infected cells and uninfected cells in a random fashion (contacts are assumed to be randomly distributed). The proportion of cells that survive infection per time step is given by . The last term in the equation represents the proliferation of CD4 ^+^ T cells and the expression was adopted from [61]. Proliferation is assumed to occur at the beginning of the time step.

Equation (6) is the equation for infected cells and  is the probability that the infected cell dies naturally. Death is assumed to occur at the end of the time step, so that  is the proportion of infected cells that survive per time step. The first term on the right hand side of equation (6), gives the gain term due to infection of CD 4^+^ T cells. The expression , represents the proportion of uninfected cells that become infected per time step.

### Modelling the specific immune response

The immune system fight HIV using several mechanisms. The humoral (neutralizing antibody) response neutralize free virus, thereby reducing the amount of free virus that can attach to and infect the CD4 ^+^ T cells [62]. Antibodies are not cells but molecules secreted by the B cells and each B cell secrete a unique antibody. Cell-mediated immunity involves the production of cytotoxic T-lymphocytes (CTLs). CTLs either release substances which kill the infected cells through a process called lysis [63] or secrete cytokines that trigger a reaction inside the infected cells that prevents the viral genome from being expressed [64, 65] and/or chemokines that inhibit viral entry into cells [66]. The lysing of infected cells will be referred to as the lytic antiviral response and the mechanism of secreting cytokines to counter the infection will be referred to as the cytokine antiviral response and the mechanism of secreting chemokines will be referred to as the chemokine antiviral response in the subsequent sections. The model which includes specific immune responses takes the form.
7891011121314

We have assumed a predator prey relationship where there is a hunt and kill relationship such as neutralizing antibodies eliminating/killing virus particles, CTLs hunting and killing the infected cells. In such relationships there is an exponential decay of the prey and an exponential increase in the predator therefore the primary reason for the exponential term selection. However we opted for a saturating type term in the case of an immune mechanism function depending on the amount (density/level) of specific cytokines or chemokines or a hybrid of saturation term in an exponential term in which case the decay depends on the density of the biological variables. For example, chemokines inhibit viral replication by blocking the critical interaction between coreceptors and the V3 domain of the viral envelope glycoprotein gp120 [66]. The infectivity of the virus is related to the number of envelope glycoproteins on its surface [67], so we assumed that the relationship is saturating because as soon as all the glycoproteins are interfered with, increasing the chemokines will not have any impact. The model is built on the assumption that the higher the levels of CTLs secreting these chemokines, the lower the infectivity of the virus. In the presence of the chemokine secreting CTLs (chemokine immune response), a virus infectivity function of the form , is assumed, where *ω* is a saturation constant. The function  as *C*_*t*_→*∞* and  as *C*_*t*_→0.

The interaction of the HIV antigen and the antibodies is similar to predation with a modification that proliferation (population growth function) of *B* cells is dependent on helper *T* cells. We thus model the interaction between neutralizing antibodies and HIV using a predator-prey relationship. We further assume that the levels of circulating antibodies is proportional to the levels of B cells. The first term on the right hand side of equation (7) is multiplied by exp(−*h*_1_*B*_*t*_), where *h*_1_ is the antibody neutralizing efficiency, to cater for the neutralizing effect of antibodies (predator effect), as *B*_*t*_→*∞*, exp(−*h*_1_*B*_*t*_)→0. It should also be noted that as *B*_*t*_→0, exp(−*h*_1_*B*_*t*_)→ 1. The expression  represents HIV genome entry through cell to cell contacts. Cell-to-cell eliminates the rate limiting step of diffusion, thereby reducing the exposure time of viral particles to neutralizing antibodies and chemokines. There are conflicting reports with regards to the efficiency of antiviral responses such as neutralizing antibodies and chemokines in inhibiting cell-to-cell transmission [68–72]. In this study, we assume that cell-to-cell transmission is not affected by the extracellular environment, antibodies and the chemokine immune response in this case.

Cytokines have been reported to inhibit the viral life cycle at the transcription level [64, 65], by targeting early proviral gene expression [73]. We therefore assume that these cytokines affect the transition probability from the first pseudo provirus stage to the second pseudo provirus stage (*Q*). It is still unclear how the CD 8^+^ T cells specifically affect HIV RNA transcription at the molecular level [65], we assume that the survival probability of the *P* stage, *θ*_2_, is reduced by a factor of exp(−*hC*_*t*_) in the presence of cytokine secreting CTLs. Here we again assume a predator-prey relationship between cytokines and HIV genes. The factor is depended on the levels of circulating CTLs such that *C*_*t*_→0, exp(−*hC*_*t*_)→1 and *C*_*t*_→*∞*, exp(−*hC*_*t*_)→0.

CTLs release proteins (perforin and granzymes) that kill infected cells [63]. The right hand side of equation (12) is multiplied by exp(−*h*_2_*C*_*t*_) to cater for the killing of infected cells by CTLs. The killing of infected cells is dependent on the levels of circulating CTLs.

Equation (13) models the dynamics of CTLs at time *t*+1. The function *f*(*T*_*t*_), represents the proliferation term for CTLs. Proliferation of CTLs is dependent on CD4 ^+^ T cell density. The higher the density of CD4 ^+^ T cells, the higher the proliferation for CTLs. We propose a function of the form, , where *χ* is the probability that proliferation occurs. The interaction between infected cells and the CTLs is modelled using a predator-prey relationship. The proportion of infected cells that have been preyed on is represented by 1− exp(−*h*_2_*C*_*t*_) so that  gives the gain term of CTLs due to interactions/contacts with infected cells. The parameter *μ*_*c*_ is the proportion of CTLs that die naturally. Death is also assumed to occur at the end of the time step.

The last equation models the dynamics of the antibodies. The function , represents proliferation of B cells and *ψ* is the probability that proliferation occurs. The proliferation term is dependent on the density of helper T cells. The interaction between antibodies and the virus is modelled using a predator-prey relationship. The proportion of virus that have been preyed on is given by 1− exp(−*h*_1_*B*_*t*_) so that the gain term of antibodies is given by *g*(*T*_*t*_)*V*_*t*_(1− exp(−*h*_1_*B*_*t*_)). The parameter *μ*_*B*_, gives the proportion of *B* cells that die naturally per time step. Death is assumed to occur at the end of the time step.

### Model analysis

#### Boundedness of solutions

An important qualitative property of the discrete dynamical system (7)-(14) relates to the boundedness and positivity of solutions, since negative solutions do not have biological meaning. We thus state the following proposition.

##### **Proposition1**

Solutions of the system of equations (7)-(14) remain non-negative and are bounded whenever |*ν*+*a*|<1.

##### Proof

From equation (11) we have


The solution of


is given by


Since  is a geometric series we have


This means that
15

It can then be shown that
16

From equation (10), it can be deduced that


Using the same procedure as above, we have


It can then be shown that


From equation (13), we have


The solution of


is given by


It can be deduced that


Using a similar procedure, it can be deduced that


The equations (7)-(10) for the virus at its different stages can be given in matrix form as


where


Let  then . Observe the following inequalities:  and *θ*_2_ exp(−*hC*_*t*_)≤*θ*_2_, and define


where inequalities hold componentwise. The solution of  is given by  This implies that


We thus have


The system of equations (7)-(14) is thus bounded whenever |*ν*+*a*|<1. □

#### Disease free equilibrium point

The system of equations (7)-(14) has a disease free equilibrium point given by


where  The following theorem is used to prove the stability of *E*_0_.

##### **Theorem2**

Let  be a fixed point of the system of equations (7)-(14).

- ● If all the eigenvalues of the Jacobian matrix of the system of equations (7)-(14) evaluated at , , have moduli strictly less than 1,  is asymptotically stable.
- ● If at least one eigenvalue has modulus greater than 1, then  is unstable.
- ● If no eigenvalue of  is outside the unit circle but at least one is on the boundary (has a modulus of 1), then , maybe stable, asymptotically stable or unstable.

##### **Proposition3**.

*E*_0_ is asymptotically stable if


##### *Proof*.

The Jacobian matrix of the system of equations (7)-(14) evaluated at *E*_0_ is given in block form as


The eigenvalues are  and . All eigenvalues has moduli less 1 if  and . Thus *E*_0_ is asymptotically stable if  and . □

#### Endermic equilibrium point

Due to the complexity of the system of equations (7)-(14), the conventional methods for finding the interior equilibrium point fails, however, we can be guaranteed that it exist by the theory of persistence and permanence of discrete dynamical systems. Persistence conditions ensure that no species will go extinct in a system of interacting species whilst permanence conditions guarantees that the size of each population is bounded and that each population settles above certain threshold values. The following are the definitions of persistence and permanence of the system of equations (7)-(14).

##### **Definition4**

[74]

1. The system of equations (7)-(14) is strongly persistent at time *τ* if  for each *t*=0,1,2,⋯*τ*.
2. The system of equations (7)-(14) is weakly persistent at time *τ* if  for each *t*=0,1,2,⋯*τ*−1 and , *D*_*τ*_+*P*_*t*_+*Q*_*τ*_+*V*_*τ*_,>0 and *T*_*τ*_>0.
3. The system of equations (7)-(14) is strongly persistent if it is strongly persistent at time *τ* for *τ*=0,1,2⋯ and .
4. The system of equations (7)-(14) is uniformly persistent if it is strongly persistent at time *τ* for *τ*=0,1,2⋯ and there exist a positive constant *ϖ* such that .
5. The system of equations (7)-(14) is permanent if it is uniform persistent and point dissipative.

The following theorem will be used to study persistence and permanence of the system of equations (7)-(14).

##### **Theorem5**.

[75] Let *E* be a locally compact space with metric *d* and let *X* be a closed subset of *E* with nonempty boundary *Y* and nonempty interior . Suppose that *f* is a continuous map on *X* with *f*(*Y*)⊂*Y* and *f*(*X*∖*Y*)⊂*X*∖*Y*. Then *f* is uniformly persistent if the following hold

1. *f* is dissipative,
2. *f*|_*Y*_ is isolated,
3. *f*|_*Y*_ is acyclic,
4. for each *M*_*i*_∈*M*, .

##### **Theorem6**.

The system equations (7)-(14) is uniformly persistent provided that


##### *Proof*.

Let *E*=*R*^*n*^ and . The system of equations (7-14) can be written as map defined as  such that


where *x*=(*D*,*P*,*Q*,*V*,*T*,*T*^∗^,*C*,*B*).

Condition 1 of Theorem 5 holds by Proposition 1 above. We now show condition 2. Define


and let *Y*=*C*. There is one constant solution *M* given by *D*=*P*=*Q*=*V*=*T*^∗^=*C*=*B*=0 and  where  is fixed point of . If  is a solution of the system of equations emanating from C, then by Proposition 3, . The set C is invariant and isolated. Thus we can conclude that *F*|_*Y*_ is isolated and acyclic. To prove condition 4, we need to show that . Suppose that , let  be a positive orbit in  such that


Let *t*_0_ be sufficiently large such that  for *ε* sufficiently small and *t*>*t*_0_. For *t*>*t*_0_, we have
17181920212223

Consider the system
24252627282930

The Jacobian matrix of the system of equations (24)-(30) at fixed point (0,0,0,0,0,0,0) is given by , where , , and .


is nonnegative and irreducible and thus it has an eigenvalue that is greater than 1 and a corresponding eigenvector which we denote by **v**. The trivial equilibrium point is thus unstable. Next we choose any number *l*>0, that is small such that


If  is a solution of the system of equations (24)-(30) with  then we are guaranteed that


From proposition 1 we have that  are bounded. It can easily be shown that the system of equations (24)-(30) is also bounded. It follows from Lemma 5.2 in [74] that the map defined by the system of equations (24)-(30) converge to a unique equilibrium point  where . This then implies that


componentwise. This contradicts the assumption that . Hence . Thus the system of equation (7)-(14) is uniformly persistent. □

##### **Proposition7**.

The system of equations (7)-(14) is permanent.

##### *Proof*.

The result follows from definition 1.5. □

## Electronic supplementary material

Additional file 1: **The first section of the file gives the elasticity analysis of the viral levels to HIV life cycle parameters.** The general trend that was observed was that the viral levels were more elastic to parameters of the late stages of the life cycle (*ϕ*, *θ*_2_)than parameters of the early stages of the viral life cycle (*β*_1_, *θ*_1_). Targeting the late stages of the HIV life cycle results in lower viral levels than targeting the early stages. The second section of the file deals with finding the form of transmission that is more efficient in vivo between cell free and cell associated transmission using mathematical models. The model results showed that cell-associated transmission is more efficient in transmitting the infection than cell-free transmission in the blood. (PDF 72 KB)

Additional file 2: **The first section of the file gives the derivation of the elasticities of the viral levels to immune response parameters formulae and the last section shows how the Negative Binomial Distribution was used to approximate the provirus stage duration.** (PDF 71 KB)
