# Supplementary material for: A comparison of elasticities of viral levels to specific immune response mechanisms in human immunodeficiency virus infection
Source: BMC Res Notes. 2014 Oct 20;7:737. doi: 10.1186/1756-0500-7-737 (PMC4221687; doi:10.1186/1756-0500-7-737)
Supplement: Supplementary file 1 — Additional file 1: The first section of the file gives the elasticity analysis of the viral levels to HIV life cycle parameters. The general trend that was observed was that the viral levels were more elastic to parameters of the late stages of the life cycle (ϕ, θ 2)than parameters of the early stages of the viral life cycle (β 1, θ 1). Targeting the late stages of the HIV life cycle results in lower viral levels than targeting the early stages. The second section of the file deals with finding the form of transmission that is more efficient in vivo between cell free and cell associated transmission using mathematical models. The model results showed that cell-associated transmission is more efficient in transmitting the infection than cell-free transmission in the blood. (PDF 72 KB) [file 13104_2014_3284_MOESM1_ESM.pdf]

Additional file 1

S.P. Showa, F. Nyabadza, S.D. Hove-Musekwa, G. Magombedze.

Elasticities of viral levels to life cycle parameters

Elasticity analysis of the viral levels to the viral life cycle parameters is performed in this section. The elasticity of the viral levels to the parameter vector $\theta = [\ \beta_1 \quad \beta_2 \quad \theta_2 \quad \theta_2 \quad \theta_3 \quad \phi \quad \mu_{T^*}\ ]$ is given by

$$\frac{dN(t+1)}{d\theta'} = (N(t)' \otimes I)\left(\frac{\partial vecA}{\partial\theta'} + \frac{\partial vecA}{\partial n(t)'}\frac{dn(t)}{d\theta'} + \frac{\partial vecA}{\partial C(t)}\frac{dC(t)}{d\theta'} + \frac{\partial vecA}{\partial B(t)}\frac{dB(t)}{d\theta'}\right)$$

$$+ (N(t)' \otimes I)\left(\frac{dvecB}{d\theta'}\right) + A\frac{dN(t)}{d\theta'} + B\frac{dn(t)}{d\theta'}.$$

We also have that

$$\frac{dn(t+1)}{d\theta'} = (n(t)' \otimes I)\left(\frac{\partial vecU}{\partial\theta'} + \frac{\partial vecU}{\partial N(t)'}\frac{dN(t)}{d\theta'}\right)$$

$$+ (n(t)' \otimes I)\left(\frac{\partial vecU}{\partial n(t)'}\frac{dn(t)}{d\theta'} + \frac{\partial vecU}{\partial C(t)'}\frac{dC(t)}{d\theta'}\right) + U\frac{dn(t)}{d\theta'},$$

$$\frac{dC(t+1)}{d\theta'} = \frac{\partial c}{\partial n(t)}\frac{dn(t)}{d\theta'} + \frac{\partial c}{\partial C(t)}\frac{dC(t)}{d\theta'}$$

and

$$\frac{dB(t+1)}{d\theta'} = \frac{\partial b}{\partial N(t)}\frac{dN(t)}{d\theta'} + \frac{\partial b}{\partial n(t)}\frac{dn(t)}{d\theta'} + \frac{\partial b}{\partial B(t)}\frac{dB(t)}{d\theta'}.$$

The elasticities are shown in Figure 1.

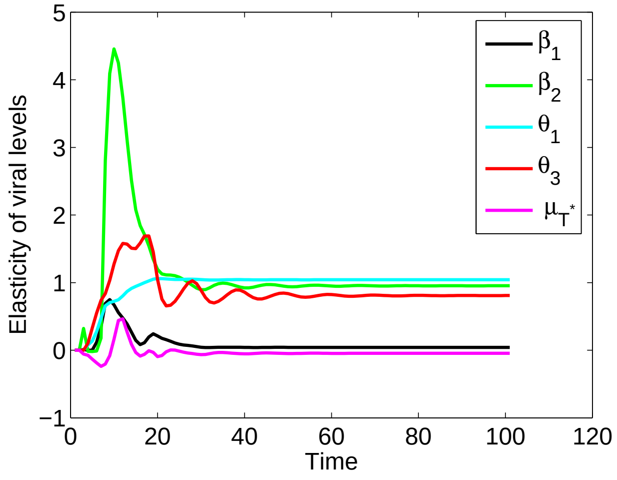

(a)

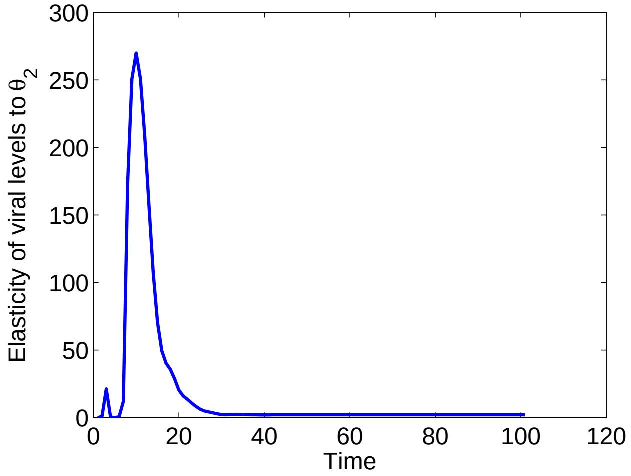

(b)

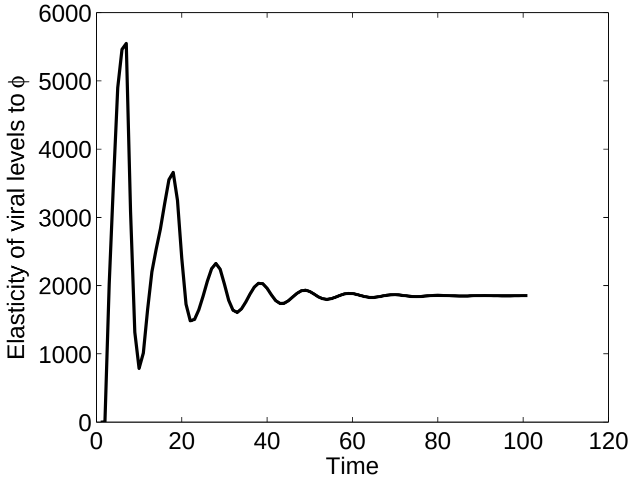

(c)

Figure 1: Elasticities of the viral levels to life cycle parameters. The implication for this result is that the viral levels are most sensitive to viral production per cell per unit time and least sensitive to the rate at which cells are infected by cell free virus.

The viral levels are most elastic to ϕ followed by θ_2 and least elastic to β_1.

Transmission efficiency of HIV in the blood

The parameter β_1 was obtained from [1]. This reference gives details on how this parameter value was computed. It was shown in the study [2], that the infectivity of the cell-associated virus is 10^2 to 10^3 times greater than the infectivity of free virus stocks so we multiplied the infectivity of the free virus by values in the ranges 10^2 to 10^3 to get the infectivity of the cell associated virus (β_2). However this range is from an in vitro model. In order to find the form of transmission that is more efficient in the blood and support the use of the values obtained from an in vitro model, we conducted a study using our mathematical models to find the form of transmission that is more efficient in the blood and simulations from these models are given below.

Cell-free transmission model

These results are obtained from a model that considers cell-free transmission whereby health cells are infected through contacts with free virus particles only i.e $\beta_2 = 0$. Results in Figure 2 suggest that cell-free virus does not spread efficiently since the viral levels are approaching zero with time. This result is consistent with results obtained in cell cultures that cell-free virus is not efficient in spreading an infection [3, 4, 5, 6].

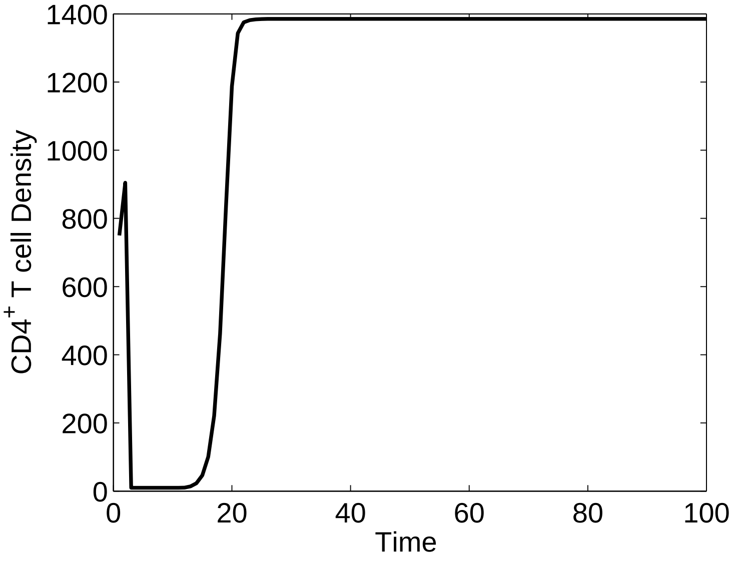

(a) Uninfected cell levels

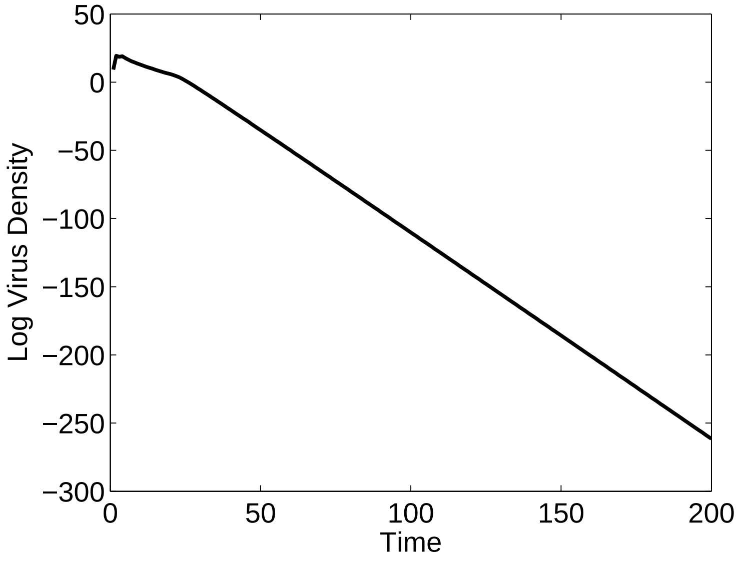

(b) Viral levels

Figure 2: The graphs gives the simulations of the cell-free transmission model. The viral levels approach zero and the $CD4^+$ T cell populations converge to a non-zero steady state as time increases. The infection dies out on its own.

Cell-to-cell transmission model

These results are obtained from a model where it is assumed that infection is through cell-associated transmission only i.e $\beta_1 = 0$.

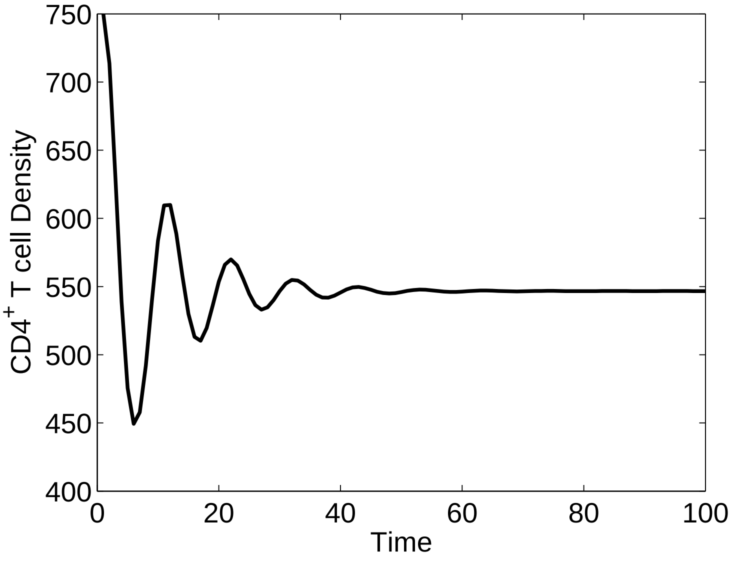

(a) Uninfected cell levels

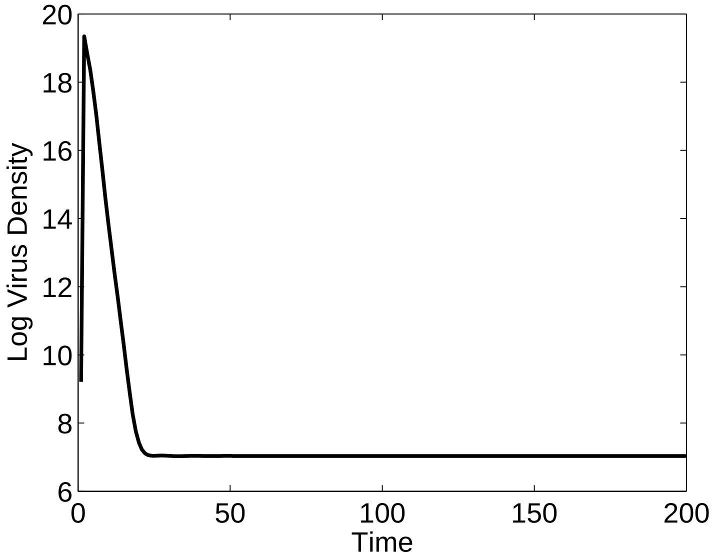

(b) Viral levels

Figure 3: The plots gives the simulations of the cell-associated transmission model. The viral and the $CD4^+$ T cell levels converge to non-zero steady states. Transmission through cell-associated virus will result in an endermic equilibrium state.

Cell-associated transmission can spread the infection efficiently as shown in Figure 3. In the cell-associated transmission model, the $CD4^+$ T cell levels stabilise slightly above 500 cells per ml of blood and the viral levels stabilise in the ranges close to 10^4 particles per ml of blood, ranges that agree with observed data.

These model results showed that cell-associated transmission is more efficient in transmit-

ting the infection than cell-free transmission in the blood, a result consistent with in vitro models [4, 5, 6]. Cell-free transmission could initiate an infection as depicted by an initial fall in $CD4^+$ T cells, however the infection does not spread efficiently. We therefore used the ranges obtained from the in vitro model to do our simulations.

References

[1] Kirschner D: **Using Mathematics to understand HIV immune dynamics**. *Notices Amer Math Soc* 1996, **43**(Suppl 2):193-202

[2] Dimitrov DS, Willey RL, Sato H, Chang LJ, Blumenthal R, Martin MA: **Quantitation of human immunodeficiency virus type 1 infection kinetics**. *J Virol* 1993, **67**(Suppl 4):2182-2190.

[3] Sourisseau, M, Sol-Foulon, N, Porrot F., Blanchet, F., and Schwartz, H: **Inefficient human immunodeficiency virus replication in mobile lymphocytes.** *J Virol* 2007, **81**(Suppl 2):1000-1012.

[4] Sato H, Orestein J, Dimitrov D, Martin M: **Cell to cell spread of HIV occurs within minutes and may not involve virus particles.** *Virology* 1992, **186:**712-724.

[5] Mothes W, Sherer NM, Jin J, Zhong P: **Virus cell-to-cell transmission.** *J Virol* 2010, **84**(Suppl 17):8360-8368.

[6] Car JM, Hocking H, Li P, Burrel C: **Rapid and efficient cell-to-cell transmission of human immunodeficiency virus infection from monocyte-derived macrophages to peripheral blood lymphocytes.** *Virology* 1999, **265:**319-329.
