# Supplementary material for: A comparison of elasticities of viral levels to specific immune response mechanisms in human immunodeficiency virus infection
Source: BMC Res Notes. 2014 Oct 20;7:737. doi: 10.1186/1756-0500-7-737 (PMC4221687; doi:10.1186/1756-0500-7-737)
Supplement: Supplementary file 2 — Additional file 2: The first section of the file gives the derivation of the elasticities of the viral levels to immune response parameters formulae and the last section shows how the Negative Binomial Distribution was used to approximate the provirus stage duration. (PDF 71 KB) [file 13104_2014_3284_MOESM2_ESM.pdf]

Additional file 2

S.P. Showa, F. Nyabadza, S.D. Hove-Musekwa, G. Magombedze.

Elasticity Analysis

The system of equations (7)-(14) can be written as

$$\begin{aligned} N(t+1) &= A\left(\theta, n(t), B(t), C(t)\right) N(t) + Bn(t), \\ n(t+1) &= U\left(\theta, N(t), n(t), C(t)\right) n(t) + S, \\ C(t+1) &= c\left(\theta, n(t), C(t)\right), \\ B(t+1) &= b\left(\theta, N(t), n(t), B(t)\right), \end{aligned} \tag{1}$$

where

$$A\left(\theta, n(t), B(t), C(t)\right) = \begin{pmatrix} 0 & 0 & 0 & \frac{\beta_1 \exp(-h_1 B_t)}{1+\omega C_t} \\ \theta_1 & \theta_3 & 0 & 0 \\ 0 & \theta_2 \exp(-hC_t) & \theta_3 & 0 \\ 0 & 0 & \theta_2 \phi T_t^* & 0 \end{pmatrix},$$

$$B = \begin{pmatrix} 0 & \beta_2 \\ 0 & 0 \\ 0 & 0 \\ 0 & 0 \end{pmatrix}, \ N(t) = \begin{pmatrix} D_t & P_t & Q_t & V_t \end{pmatrix}', \ n(t) = \begin{pmatrix} T_t & T_t^* \end{pmatrix}',$$

$$U\left(\theta, N(t), n(t), C(t)\right) = \begin{pmatrix} H & 0 \\ K & (1-\mu_{T^*})\exp(-h_2 C_t) \end{pmatrix},$$

$$\text{where } \mathrm{H} = \nu \exp\left(-\frac{\beta_1 \mathrm{V_t}}{1+\omega \mathrm{C_t}} - \beta_2 \mathrm{T_t^*}\right) + \mathrm{a}\exp\left(-\frac{\mathrm{T_t}}{\mathrm{K}}\right)$$

$$\text{and } \mathrm{K} = \left[1 - \exp\left(-\frac{\beta_1 \mathrm{V_t}}{1+\omega \mathrm{C_t}} - \beta_2 \mathrm{T_t^*}\right)\right]\exp(-\mathrm{h_2 C_t}),$$

$$c\left(\theta, n(t), C(t)\right) = f(T_t) T_t^* \left[1 - \exp(-h_2 C_t)\right] + (1-\mu_C) C_t,$$

$$b\left(\theta, N(t), n(t), B(t)\right) = g(T_t) V_t \left[1 - \exp(-h_1 B_t)\right] + (1-\mu_B) B_t,$$

$\theta = \left(\begin{array}{cccccc} h & h_1 & h_2 & \mu_{T^*} & \chi & \psi \end{array} \right), B(t) = B_t,\ C(t) = C_t$, and $S = \left(\begin{array}{cc} S_T & 0 \end{array} \right)'$ Taking the differentials of the first equation of (1) we have

$$dN(t+1) = (dA)N(t) + A(dN(t)) + Bdn(t) + (dB)n(t), \tag{2}$$

where $A(\theta, n(t), B(t), C(t)) = A$. Multiplying the first entry on the right hand side of equation (2) by a 4×4 identity matrix I, respectively, we have

$$dN(t+1) = I(dA)N(t) + A(dN(t)) + Bdn(t). \tag{3}$$

Applying the *vec* operator to (3) we have

$$\begin{aligned} dN(t+1) &= (N(t)' \otimes I)\, dvecA + AdN(t) && (4) \\ &\quad + Bdn(t), \\ &= (N(t)' \otimes I) \left(\frac{\partial vecA}{\partial \theta'} d\theta + \frac{\partial vecA}{\partial n(t)'} dn(t) + \frac{\partial vecA}{\partial C(t)} dC(t) \right) && (5) \\ &\quad + (N(t)' \otimes I) \frac{\partial vecA}{\partial B(t)} dB(t) + AdN(t) + Bdn(t). \end{aligned}$$

Multiplying the right hand side of equation (5) by the identity $\frac{d\theta}{d\theta'}$ we get

$$\begin{aligned} dN(t+1) &= (N(t)' \otimes I) \left(\frac{\partial vecA}{\partial \theta'} + \frac{\partial vecA}{\partial n(t)'} \frac{dn(t)}{d\theta'} + \frac{\partial vecA}{\partial C(t)} \frac{dC(t)}{d\theta'} \right) d\theta \\ &\quad + (N(t)' \otimes I) \left(\frac{\partial vecA}{\partial B(t)} \frac{dB(t)}{d\theta'} \right) d\theta + A \frac{dN(t)}{d\theta'} d\theta + B \frac{dn(t)}{d\theta'} d\theta. \end{aligned}$$

by the First identification theorem which states that if $dy = Qdx$ then $\frac{dy}{dx} = Q$ we have,

$$\frac{dN(t+1)}{d\theta'} = (N(t)' \otimes I) \left(\frac{\partial vecA}{\partial \theta'} + \frac{\partial vecA}{\partial N(t)'} \frac{dN(t)}{d\theta'} + \frac{\partial vecA}{\partial n(t)'} \frac{dn(t)}{d\theta'} + \frac{\partial vecA}{\partial C(t)} \frac{dC(t)}{d\theta'} \right)$$

$$+ (N(t)' \otimes I) \left(\frac{\partial vecA}{\partial B(t)} \frac{dB(t)}{d\theta'} \right) + A \frac{dN(t)}{d\theta'} + B \frac{dn(t)}{d\theta'}$$

Performing the same operations on the second equation of (1) we have,

$$\frac{dn(t+1)}{d\theta'} = (n(t)' \otimes I) \left(\frac{\partial vecU}{\partial \theta'} + \frac{\partial vecU}{\partial N(t)'} \frac{dN(t)}{d\theta'} \right)$$

$$+ (n(t)' \otimes I) \left(\frac{\partial vecU}{\partial n(t)'} \frac{dn(t)}{d\theta'} + \frac{\partial vecU}{\partial C(t)'} \frac{dC(t)}{d\theta'} \right) + U \frac{dn(t)}{d\theta'}.$$

It can easily be shown that

$$\frac{dC(t+1)}{d\theta'} = \frac{\partial c}{\partial \theta'} + \frac{\partial c}{\partial n(t)} \frac{dn(t)}{d\theta'} + \frac{\partial c}{\partial C(t)} \frac{dC(t)}{d\theta'}$$

and

$$\frac{dB(t+1)}{d\theta'} = \frac{\partial b}{\partial \theta'} + \frac{\partial b}{\partial N(t)} \frac{dN(t)}{d\theta'} + \frac{\partial b}{\partial n(t)} \frac{dn(t)}{d\theta'} + \frac{\partial b}{\partial B(t)} \frac{dB(t)}{d\theta'}.$$

Given initial conditions, we can recursively compute $N(t)$, $B(t)$, $C(t)$, $n(t)$, $\frac{dN(t)}{d\theta'}$, $\frac{dB(t)}{d\theta'}$, $\frac{dC(t)}{d\theta'}$ and $\frac{dn(t)}{d\theta'}$. The mature virus population is given by weighted sum of stage densities as

$$V(t) = e_4' N(t),$$

where e_4 is column vector with a one on the fourth position and zeros everywhere. The sensitivity of the virus population to the vector of parameters is then given by

$$\frac{dV(t)}{d\theta'} = e_4' \frac{dN(t)}{d\theta'}.$$

Proportional changes known as elasticities are used to compare the parameter sensitivities. The elasticity of the virus population $V(t)$ to the parameter vector θ is given by

$$\frac{\theta}{V(t)} \frac{dV(t)}{d\theta'}.$$

Negative binomial distribution method to estimate transition probabilities

To calculate the proportion that goes to the virus stage and the proportion that remains in the provirus stage, we divide the provirus stage into K identical pseudo stages and let the probability of moving to the next stage in each of the pseudo stages be γ. The time τ required for the provirus to pass through all the K stages is the time required for the Kth success in a series of identical Bernoulli trials with probability of success γ. This time has a negative Binomial distribution with mean $\tau = \frac{K}{\gamma}$ and variance $\frac{K(1-\gamma)}{\gamma^2}$. The number of pseudo stages and their common transition probabilities are calculated from the mean and variance as;

$$\gamma = \frac{\bar{\tau}}{var(\tau) + \bar{\tau}}, \quad (6)$$

$$K = \frac{\bar{\tau}^2}{var(\tau) + \bar{\tau}}. \quad (7)$$

HIV has a life cycle of one to two days [1, 2]. The early steps of HIV replication (from entry of the virus to integration) takes one day [3]. The average duration of the provirus stage is computed as 13.6 hours with a variance of 94.8 hours. With these values and using expressions (6) and (7), $\gamma = 0.1263$ and $K = 2$. Thus one pseudo provirus stage is added to the model and is represented by Q. The transition probability within the pseudo provirus stages and between the provirus stage and the mature virus stage, is then computed as $\theta_2 = 0.5$ and $\gamma = 0.06315$. The probability that the provirus will survive and remain in the

same pseudo provirus stage is computed as $\theta_3 = 0.5(1-\gamma) = 0.43685$, where 0.5 is the stage specific survival [4].

References

[1] Folks T, Powell DM, Lightfoote MM, Benn S, Martin MA, A.S. Fauci: **Induction of HTLV-111/LAV from a non virus producing T cell line, Implications for latency**. *Science* 1986, **231:**600-602.

[2] Wong D: **Human Immunodeficiency Viruses** [http://virology-online.com/viruses/HIV.htm].

[3] Butler SC, Mark S, Hansen T, Bushman FD: **A quantitative assay for HIV DNA integration in vivo.** *Nat Med* 2001 ,**7**(Suppl 5):631-634.

[4] Brinchmann JE, Albert J, Vartal F, **Few infected CD4^{+} T cells but a high proportion of replication-competent provirus in the asymptomatic human immunodefiency virus type 1 infection.** *J Virol* 1991, **65**(Suppl 4):2019-2023.
